# Three-dimensional topology-based analysis segments volumetric and spatiotemporal fluorescence microscopy

**DOI:** 10.1017/S2633903X23000260

**Published:** 2023-12-14

**Authors:** Luca Panconi, Amy Tansell, Alexander J. Collins, Maria Makarova, Dylan M. Owen

**Affiliations:** 1 Institute of Immunology and Immunotherapy, University of Birmingham, Birmingham, UK; 2 College of Engineering and Physical Sciences, University of Birmingham, Birmingham, UK; 3 Centre of Membrane Proteins and Receptors, University of Birmingham, Birmingham, UK; 4 School of Mathematics, University of Birmingham, Birmingham, UK; 5 Department of Chemistry, University of Cambridge, Cambridge, UK; 6 School of Biosciences, College of Life and Environmental Science, University of Birmingham, Birmingham, UK; 7 Institute of Metabolism and Systems Research, College of Medical and Dental Sciences, University of Birmingham, Birmingham, UK

**Keywords:** fluorescence microscopy, cell segmentation, cell tracking, R, topological data analysis

## Abstract

Image analysis techniques provide objective and reproducible statistics for interpreting microscopy data. At higher dimensions, three-dimensional (3D) volumetric and spatiotemporal data highlight additional properties and behaviors beyond the static 2D focal plane. However, increased dimensionality carries increased complexity, and existing techniques for general segmentation of 3D data are either primitive, or highly specialized to specific biological structures. Borrowing from the principles of 2D topological data analysis (TDA), we formulate a 3D segmentation algorithm that implements persistent homology to identify variations in image intensity. From this, we derive two separate variants applicable to spatial and spatiotemporal data, respectively. We demonstrate that this analysis yields both sensitive and specific results on simulated data and can distinguish prominent biological structures in fluorescence microscopy images, regardless of their shape. Furthermore, we highlight the efficacy of temporal TDA in tracking cell lineage and the frequency of cell and organelle replication.

## Impact Statement

In this work, we introduce a 3D cell segmentation pipeline for volumetric and spatiotemporal analyses. Existing cell segmentation methods may be biased by geometric constraints or subjectivity from training data, and increased dimensionality often brings additional parameterisation. Here, we present extensions of Topological Boundary Line Estimation using Recurrence Of Neighbouring Emissions (TOBLERONE), a topological image analysis tool which identifies homological features of the image space without assuming object geometry. We extend this algorithm to incorporate three dimensions, both spatially and temporally. This allows for total image, volume and video segmentation using only a single parameter. As such, TOBLERONE provides a simple framework for segmenting cells and organelles in fluorescence microscopy, operating without the drawbacks of conventional geometric and machine learning-based analysis methods. All software has been made open-source and publicly available to support researchers across the fields of cell biology and bioinformatics.

## 1. Introduction

Fluorescence microscopy is a core technology in biological research, using chemically specific probes to fluorescently tag structures of interest and highlight the morphology and behavior of cells^(^^1^ ^,^^2^ ^)^. Advances in fluorescence microscopy have allowed for volumetric imaging and imaging with temporal resolutions, producing three-dimensional (3D) scans of cells and live-cell videos^(^^3^ ^–^^6^ ^)^. Fluorescence microscope systems output image data in which the volumetric components (pixels or voxels) containing fluorescing objects display a distinctly higher intensity than their background. Image segmentation algorithms can then separate these objects from the background^(^^6^ ^–^^8^ ^)^. Without these techniques, researchers must undertake time-consuming manual segmentation and quantification, which can lead to subjective and irreproducible results^(^^9^ ^–^^11^ ^)^. As such, they are essential for automated and objective analysis of fluorescence microscopy data^(^^9^ ^)^.

State-of-the-art segmentation algorithms rely on machine learning-based approaches, which undertake supervised learning to interpret patterns in the data corresponding to specific objects^(^^12^ ^,^^13^ ^)^. These methods have been shown to perform well on 2D data and carry the advantage that they are highly adaptable^(^^14^ ^,^^15^ ^)^. With the advent of advanced models such as Segment Anything, 2D segmentation for most projects can be produced with minimal user input^(^^16^ ^)^. However, there is no direct method of applying existing 2D machine learning algorithms to higher dimensional data sets without completely retraining a new algorithm^(^^17^ ^,^^18^ ^)^. Advances in convolutional and recurrent neural networks have shown promise for cellular and biomedical image segmentation, but are yet to attain the accuracy of 2D machine learning-based segmentation^(^^19^ ^,^^20^ ^)^.

In the absence of supervised learning, a range of classical (non-machine learning-based) methods exist for 3D image segmentation^(^^21^ ^)^. These are typically primitive, require a suitable degree of parameter estimation, and rely on simple background-foreground separation techniques, such as thresholding^(^^21^ ^,^^22^ ^)^. Such parameters are typically tuned automatically for machine learning models, and while parameter estimation is possible for some classical methods, it is not often built in^(^^23^ ^,^^24^ ^)^. Once the foreground has been isolated, segmentation must be undertaken by a separate region-based technique, such as the Watershed algorithm^(^^25^ ^,^^26^ ^)^. All-encompassing algorithms, which achieve true segmentation in a single instance, typically probe for geometric properties of the objects they are trying to identify^(^^27^ ^)^. This makes them unsuitable for segmentation of objects with atypical or unpredictable morphologies, as is often seen in biological data^(^^27^ ^)^.

In this work, we introduce two new algorithms, built on the existing topological data analysis (TDA) technique TOBLERONE, for segmentation and quantification of 3D volumetric (3DTOB) and spatiotemporal (tempTOB) data sets^(^^28^ ^)^. Topological decomposition of image intensity variation across imposed gradient fields has shown promise in both 2D and 3D segmentation of cells and organelles, especially in fluorescence microscopy^(^^29^ ^,^^30^ ^)^. Here, we devise complete algorithms for volumetric segmentation in 3D data and dynamic tracking of live-cell data. As topological methodologies, the TOBLERONE family of algorithms require no training data, are robust to imaging artifacts, can be extended to an arbitrary number of dimensions, and are invariant to variations in geometry, allowing them to function regardless of cell or organelle morphologies^(^^28^ ^,^^31^ ^–^^33^ ^)^. Furthermore, the algorithms automatically extract statistics of the volumetric properties of the underlying objects, such as size and position, and dynamic processes, such as change in the number of objects identified over time.

We apply these novel techniques to simulated spatial 3D and spatiotemporal data sets and compare sensitivity and specificity with existing 3D image segmentation methods under imposed imaging artifacts, such as noise and blur. We demonstrate the use of 3DTOB in practice by segmenting scans of Jurkat T-cells derived from 3D fluorescence microscopy. The algorithm automatically extracts meaningful image statistics to determine the geometric properties of the cells, which agree with existing literature. We then apply tempTOB in dynamic, experimentally derived live-cell data of *Schizosaccharomyces pombe* undergoing nuclear division. We find that the algorithm is capable of tracking both nuclear division and lineage.

## 2. Materials and Methods

### 2.1. Jurkat T cell culture and labelling

Jurkat T cells (ATCC TIB-152) were grown in RPMI (Sigma-Aldrich, Madison, WI). The culture media was supplemented with 10% fetal calf serum (FCS) (PAA), 10 mM HEPES (Sigma-Aldrich), 1 mM sodium pyruvate (Sigma-Aldrich), 2 mM L-glutamine, and antibiotics [50 units penicillin, 50 μg streptomycin and 100 μg neomycin per mL] (Sigma-Aldrich). Cells were incubated at 37 °C with 5% CO_2_. Labeling was done by resuspending 1 mL of cells in a solution of Nuclear Mask DeepRed [1 in 200 dilutions from stock (250× concentrate in DMSO)] (H10294, ThermoFisher) and WGA-AlexaFluor 488 [10 μg/mL concentration] (W11261, ThermoFisher). They were then incubated for 15 minutes at 37 °C before being washed 3x in PBS. For fixation, cells were resuspended in 4% PFA and incubated at 37 °C for 15 minutes, before being washed 3x in PBS. 3D stack was recorded in each color channel using a Zeiss LSM 900 confocal in confocal scan mode (voxel size: 0.62 *×* 0.62 × 0.54 μm (*xyz*)).

### 2.2. *S. pombe* growth, strain generation, and imaging

*Schizosaccaramyces pombe* was grown in YES medium. Media and genetic techniques follow protocols listed in literature^(^^34^ ^)^. The *S. pombe* strain expressing Bop1-mCherry was obtained via genetic transformation of wild-type strain using homologous recombination. Plasmids containing mCherry gene and 3’UTR fragment of bop1 gene (SPAP32A8.03c) were constructed via standard molecular biology techniques. The endogenous bop1 gene was tagged with mCherry maintaining all native regulatory elements. The transformation was performed using the lithium acetate-based method described in literature^(^^34^ ^)^. Fluorescent microscopy images were generated using a Zeiss Axiovert 200 M microscope with a Plan Apochromat 100X, 1.4 NA objective. The microscope was equipped with an UltraView RS-3 confocal system, including a CSU21 confocal optical scanner, a 12-bit digital cooled Hamamatsu Orca-ER camera (OPELCO, Sterling, VA), and a krypton-argon triple line laser illumination source. Image stacks were acquired with seven sections spaced 0.5 μm apart, at intervals of 1 minute. The z-stack maximum projection images were obtained using ImageJ v2.9.0^(^^35^ ^)^. Imaging was conducted on *S. pombe* cells placed in sealed growth chambers containing 2% agarose YES medium.

### 2.3. Data simulation

8-bit stacks were simulated manually in the GIMP raster graphics editor. Stacks varied between 8 and 22 frames. Each frame was 256 by 256 pixels in size, with each pixel’s brightness intensity defined between 0 and 1. Prominent objects were defined at grayscale intensities between 0.5 and 1 over a background defined at 0. Further manipulation of each image was performed in ImageJ. Gaussian blur (with standard deviations *σ*_*1*_ = 0, 5, 10) was applied laterally to each frame (and axially, for volumes), then overlaid with Gaussian noise (with standard deviations *σ*_*2*_ = 0, 5, 10). To recapitulate experimental results, all images were subject to simple denoising by Gaussian smoothing proportional to the degree of noise. Resulting images were normalized to return intensity values to the range of 0 to 1. Five images were considered over nine image quality conditions for both z- and t-stacks, giving a total of 90 simulated images. See the Data Availability Statement for all simulations, including variations in image quality.

### 2.4. Segmentation and tracking software

The TOBLERONE software package v1.1.0 was written in the R programming language v4.2.0 and employed in the integrated development environment RStudio, version 2022.07.1 + 554, and is available for use under GNU General Public License v3.0. See Supplementary Material for the code. 3D Simple Segmentation and 3D Spot Segmentation were undertaken in ImageJ using the 3DSuite plugin v4.0.93^(^^36^ ^)^. Suitable input parameters were determined iteratively for all classical algorithms. Trackmate v7.11.1 was applied in ImageJ using pre-defined masks from TOBLERONE to assign track lines^(^^37^ ^,^^38^ ^)^.

## 3. Results

### 3.1. 3D volumetric segmentation by 3D TOBLERONE

The 3D implementation of TOBLERONE takes as input: a stack of images, where each voxel represents the intensity of fluorescence detected in each volumetric component, and a persistence threshold, τ, which determines the algorithm’s sensitivity to overlapping objects. The raw output of 3D fluorescence microscopy provides the image stack, which can be fed directly into 3DTOB. However, depending on the quality of the images, pre-processing may first be required to normalize brightness intensity and apply light denoising. This helps to highlight the prominent topological features of the image space so they may be more easily detected. The algorithm itself exploits the principles of persistent homology, which constructs a gradient field subject to a pre-defined metric to identify basins of attraction, which typically signify the existence of an object^(^^39^ ^)^. Persistent homology, like all gradient-based methods, usually requires the input of a density field, which must be constructed externally. For fluorescence microscopy, this density can be interpreted as the literal density of photons emitted by fluorescent probes across the ROI, which is represented in the grayscale image as the intensity of each pixel^(^^40^ ^)^.

The densest regions of the image space are taken to be the origin point for identifiable objects, which are then constructed iteratively^(^^41^ ^)^. For 3D image analysis, this is done by ordering the intensity of each pixel across each frame, producing what is known as a filtration scheme^(^^40^ ^,^^42^ ^)^. Objects are identified by iteratively analyzing each entry in the filtration to determine local maxima, then sequentially attaching pixels to their neighbors across multiple adjacent frames^(^^43^ ^)^. The brightest pixel of each object is designated as the root – the source of the object – to which any other pixel may be attached provided its intensity is no less than τ different from the root’s^(^^44^ ^)^. A series of connected components is gradually constructed in the image space. If two separate connected components intersect, they may be aggregated into one provided the difference between the roots is no greater than τ. At the conclusion of the process, any object whose root has an intensity less than τ is also filtered out to remove background. The result of the algorithm is a list of separate connected components corresponding to regions of continuous intensity, which represent fluorescing biological structures. Spatially descriptive statistics are calculated and output automatically, revealing crucial information about the size, position, and intensity of each structure identified.

**Figure 1.**
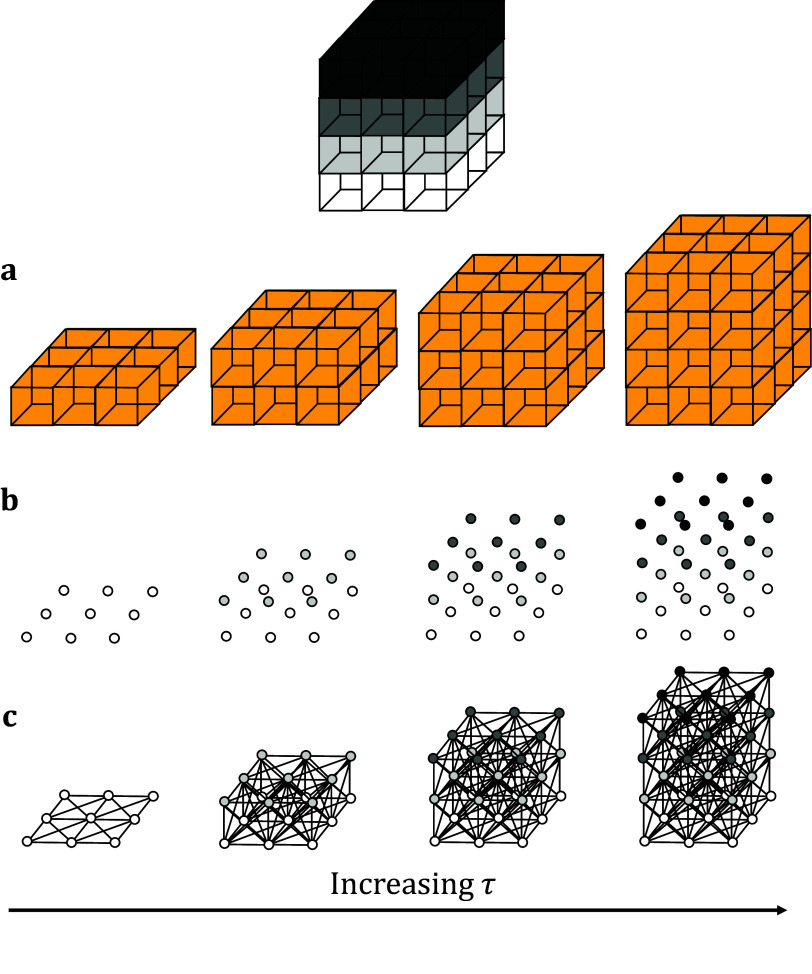
Topological representations of a z-stack. (a) Stacks may be binarized by thresholding so that specific voxels become activated. (b) Each voxel represents a point in 4D grayscale color-space. (c) The network representation establishes connectivity between neighboring active voxels. Increasing the persistence threshold permits connection of lower-intensity voxels.

This method builds upon existing planar TDA techniques, with the primary difference being that we now employ cubical complices, rather than the more commonly used simplicial homology format^(^^45^ ^)^. This change in homology better lends itself to the coarse data structure of images and stacks. As with its 2D counterpart, the optimal persistence threshold can be iteratively estimated by initializing at τ = 0.5 and altering the threshold until the returned number of connected components (that is, biological structures) matches the expected number. Persistent homology is relatively stable under small perturbations to the persistence, so minor variations to the persistence threshold will not generally alter the number of objects or their boundaries. Increasing the threshold will result in greater merging and fewer connected components while decreasing the threshold will reduce the penalty on segmentation and output more connected components. This choice of parameter will suffice across multiple data sets acquired under the same microscope conditions. As such, only one stack is required for parameter calibration (Figure 1).

### 3.2. Spatiotemporal segmentation by temporal TOBLERONE

Although structurally similar, spatiotemporal data, represented by videos, denote a vastly different data type to 3D volumetric data. Segmentation by 3D TOBLERONE is used to identify separate connected objects in 3D space and, as such, will conglomerate all voxels connected to the initial root into one object. To employ this on live-cell video data would be inappropriate as it would be unable to track the splitting (e.g. by mitotic division) or merging (e.g. by vesicle fusion) of objects that were otherwise distinct. To overcome this drawback, we implement a separate topological segmentation tool for temporal data, known as tempTOB. This variant still makes use of persistent homology but considers two spatial dimensions rather than three. Functionally, each frame in the video is considered separately and analyzed through standard 2D TOBLERONE. This determines each temporally distinct connected component and provides a list of spatial roots for each frame. While alternative tracking algorithms, such as the nearest-neighbor approach, may suffice for the purposes of establishing temporal connectivity, they may not explicitly track lineage without adaptation. Instead, we implement a topological methodology to avoid this drawback and any additional parameterization these tracking approaches may bring^(^^46^ ^)^.

**Figure 2.**
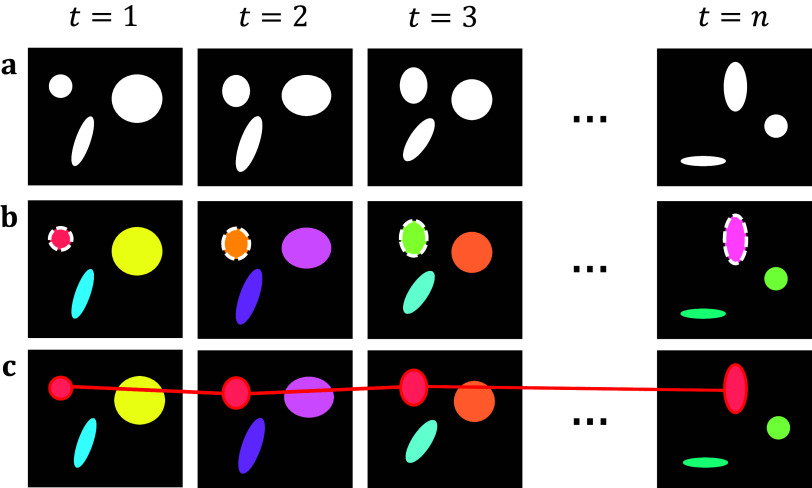
Topological decompositions of videos, or t-stacks. (a) Videos are comprised of a series of distinct frames. (b) 2D segmentation establishes connectivity within frames, but not between frames. (c) Temporal topological segmentation connects components spatially and temporally while tracking addition or deletion. An example spatiotemporal component (red) is formed from a series of spatial components.

Starting with the initial frame, we iterate over each pixel in each object and compare it with the same pixels in the following frame. If the number of unique roots is unchanged, then we assign the spatial root of the spatial object as the new spatiotemporal root of the spatiotemporal object. If we identify two or more unique roots shared among the same pixels in the following frame, we attach the object whose root has the brightest intensity to the current spatiotemporal object and create new, distinct components for all other roots. This allows for a single object in one frame to split into multiple objects in a subsequent frame. Simultaneously, we record each spatial root identified per frame, if any root is found to be connected to two separate spatiotemporal objects, then this implies that the objects have merged. As such, we take all spatiotemporal objects which share this spatial root, and preserve the one with the brightest spatiotemporal root, whilst killing off the others. Ultimately, we can use this to track individual objects across multiple frames and handle splitting and merging without aggregating all connected components into one. Separate summary statistics are provided for the size, position, intensity, and lifespan of each object as well as the change in identified objects across frames, allowing for lineage tracing (Figure 2).

### 3.3. Demonstration with simulated volumetric data

To test the performance of 3D TOBLERONE, we produced a set of simulated ground truth stacks with varied geometries and topologies (see Supplementary Material). Each stack contained an arbitrary number of 3D objects of varying intensity, and each image was segmented using 3D TOBLERONE as well as two alternative segmentation approaches, 3D Simple Segmentation and 3D Spot Segmentation^(^^36^ ^,^^47^ ^,^^48^^)^. To test the impact of image quality, the performance of all algorithms was compared across a range of image conditions. In particular, spherical Gaussian blur was simulated over each voxel in each image with standard deviations of *σ_1_* = 0, 5, 10, scaled such that axial and lateral blur were equal. Then, Gaussian noise was applied with standard deviations of *σ_2_* = 0, 5, 10. This simulated the lateral imaging artifacts often seen in experimental fluorescence microscopy. We selected this range of artifact parameters to determine the impact of image quality on segmentation. The algorithm’s sensitivity was quantified as the fraction of ground-truth foreground pixels correctly labeled as active by the algorithm, while the specificity was given by the fraction of ground-truth background pixels correctly labeled as inactive. To prevent background from skewing sensitivity and specificity, we considered voxels only within a given interval of each active ground-truth voxel comprising the original object. For 3D volumetric components, this was set to an 11 by 11 by 11 voxel grid (5 voxels in each direction) to compare the effects of both axial and lateral imaging artifacts. Additionally, we recorded the number of connected components found by each algorithm.

**Figure 3.**
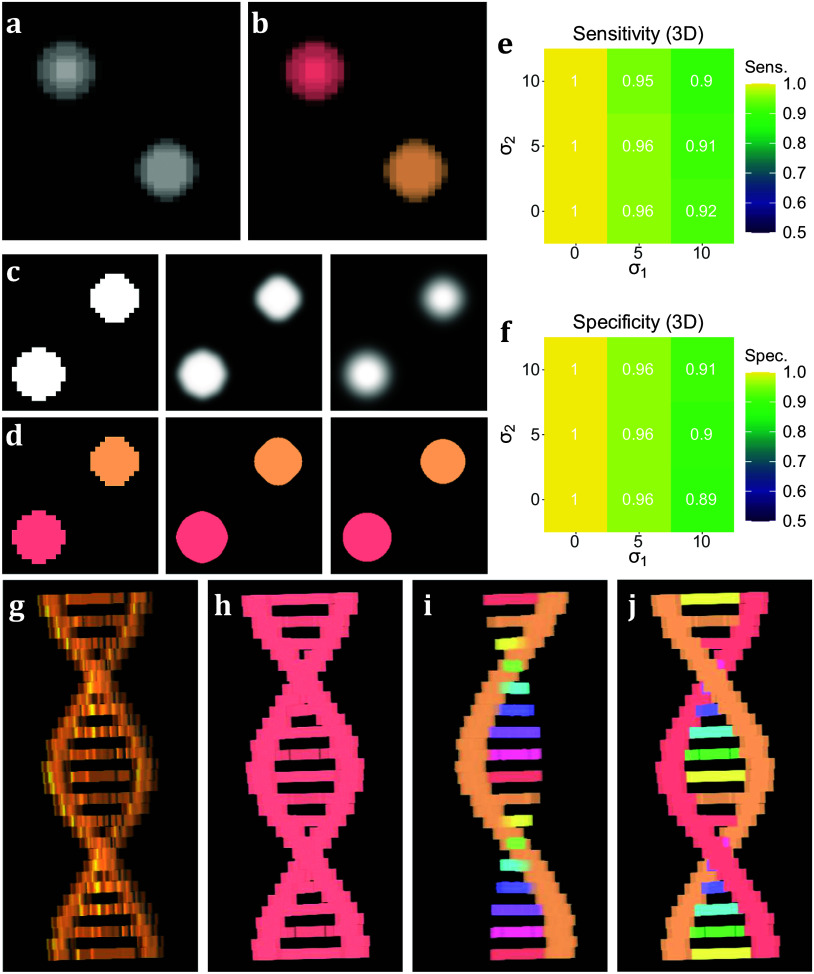
Performance on simulated data sets. (a) *xyz* projection of a simulated z-stack containing cell-like structures. (b) Segmentation of the structures as identified by 3D TOBLERONE. (c) The same slice of a simulated stack under decreasing levels of image quality. (d) Slices of the resulting 3D segmentation from reduced-quality images. (e, f) Volumetric sensitivity and specificity analysis on results of 3D TOBLERONE. (g) A simulated double helix. Branches between the two main backbone strands have a lower voxel intensity than the strands themselves. (h) Results of 3D Simple Segmentation on helix data. The entire structure is returned as one object. (i) Results of 3D Spot Segmentation on helix data. A significant portion of the object is no longer detected. (j) Results of 3D TOBLERONE on helix data. The two main backbone strands and each branch between are detected as separate objects.

On average, TOBLERONE achieved a sensitivity of 0.955 and a specificity of 0.9528 (over the total 45 images). This implies that most active voxels were correctly identified as belonging to an object and most inactive voxels were correctly considered as part of the background. The statistics surpassed their counterparts for 3D Spot Segmentation (0.7075 and 0.8862 respectively) and were on par with 3D Simple Segmentation (0.9455 and 0.9522 respectively)^(^^36^ ^)^. This suggests that TOBLERONE is capable of accurately and precisely segmenting objects, even under poor image quality, and can outperform existing classical segmentation methods. Furthermore, Figure 3e–f highlights the change in sensitivity and specificity, under the varying levels of noise and blur, on average. Results suggest that 3D TOBLERONE’s performance deteriorates as image quality worsens, and is seemingly more prone to failure with an increase in blur over noise. However, even at particularly low image quality, 3D TOBLERONE outperforms its counterparts. Of the techniques used, TOBLERONE had the highest probability to return the correct number of connected components (0.71 for TOBLERONE, 0.53 for Simple Segmentation, 0.28 for Spot Segmentation). As shown in Figure 3g–j, TOBLERONE can distinguish adjacent objects without compromising segmentation quality or permitting object loss.

### 3.4. Demonstration with simulated spatiotemporal data

**Figure 4.**
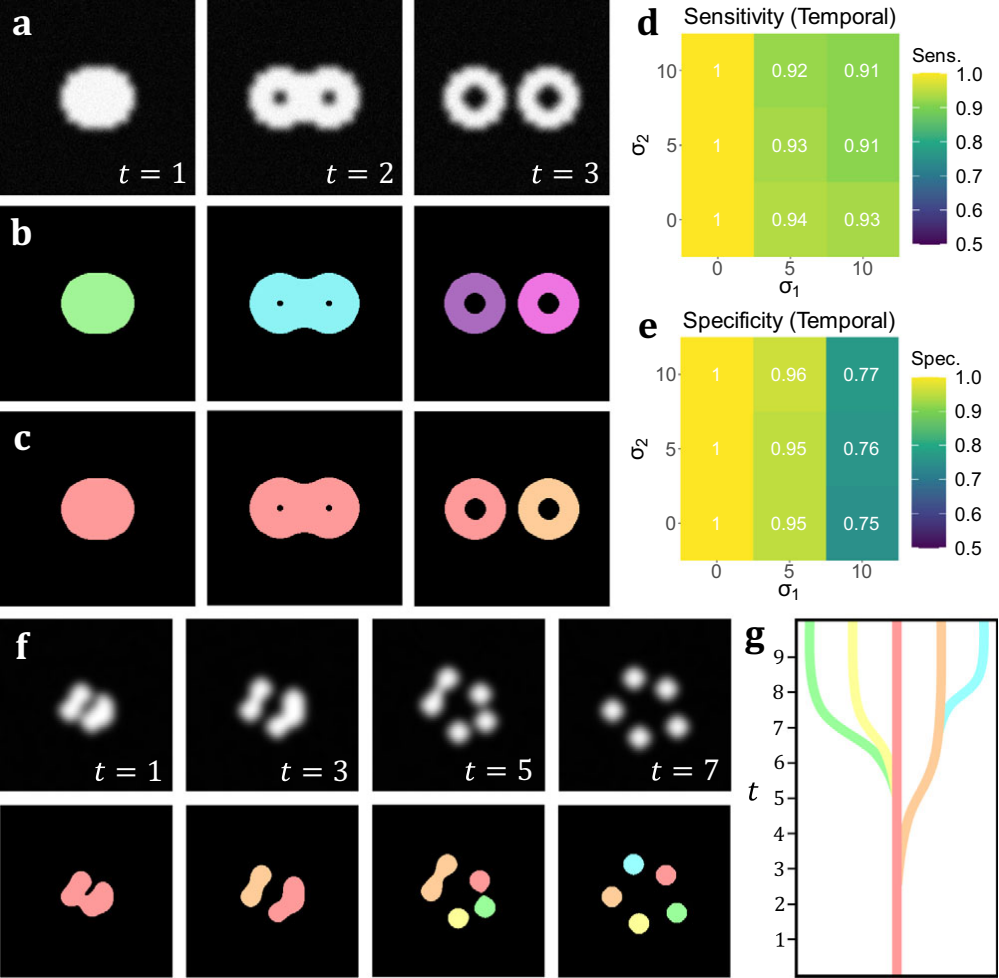
(a) A t-stack simulation of one object dividing into two. The topology and morphology of the objects change over time. (b) 2D segmentation of the t-stack. New, unconnected objects are created in each frame. (c) Temporal segmentation of the t-stack. Objects are connected across frames and a new object is created at the moment of splitting. (d, e) Spatiotemporal sensitivity and specificity analysis on results of temporal TOBLERONE. (f) Diagram of one object splitting into five across several time frames. (g) Schematic of a lineage tree of the spatiotemporal objects given in (f).

In addition to the analyses on volumetric simulations, we produced a range of videos with arbitrarily-shaped simulated objects that displayed dynamic behavior designed to test the capabilities of temporal TOBLERONE. This included the splitting and merging of objects. Unlike the 3D volumetric case, there are no pre-existing, classical video segmentation techniques for identifying objects without imposing specific geometries. That is, no video segmentation algorithm – which does not employ machine learning or permit arbitrary object morphology – exists for fluorescence microscopy. As such, while there is no basis to compare performance, we can still quantify the sensitivity and specificity of the algorithm under different image qualities. As above, we calculate the sensitivity, specificity, and number of connected components returned. For t-stacks, an 11 by 11 by 3-pixel grid was observed around each active pixel so that each frame would only be compared to the previous and subsequent frames. The average sensitivity and specificity for each combination of parameters *σ_1_*, *σ_2_* is given in Figure 4d–e. As with its volumetric counterpart, temporal TOBLERONE experiences a drop in both statistics as image quality is reduced. However, even at the lowest simulated image quality, the algorithm still achieved an average sensitivity of 0.9485 and average specificity of 0.9053. Additionally, the number of connected components identified over the entire video was recorded and always matched the number (including splits) in the ground truth data. This suggests that temporal TOBLERONE can segment dynamic objects and trace splitting or merging processes, even under poor image quality.

### 3.5. Demonstration with experimental data

**Figure 5.**
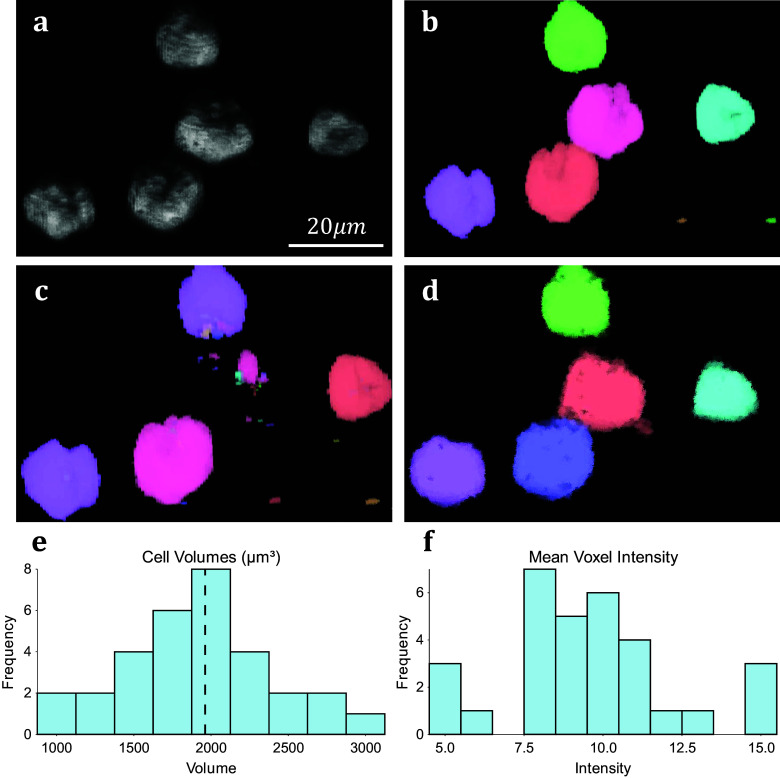
Results on experimental data. (a) 3D visualization of fluorescing Jurkat T-cells. (b) Results of 3D Simple Segmentation on cell data. (c) Results of 3D Spot Segmentation on cell data. (d) Results of 3D TOBLERONE on cell data. (e) Histogram of cell volumes identified by 3D TOBLERONE, the mean volume of 1961.6μm^3^ is signified by a dashed line. (f) Histogram of mean voxel intensity, one of several summary statistics output by 3D TOBLERONE.

We assess the performance of both methodologies qualitatively on real experimental data. For 3D volumetric data, non-activated Jurkat T-cells were cultured and labeled with Nuclear Mask DeepRed and WGA-AlexaFluor 488 before fixation with 4% PFA. A 3D stack was then recorded for each color channel using a Zeiss LSM 900 confocal microscope in confocal scan mode. These cells typically display a spherical morphology and float in suspension separately (Figure 5a), making them good candidates for testing image segmentation. 3D TOBLERONE shows appropriate discrimination against the background and can easily overcome variations in the morphology of the cells (Figure 5d). Furthermore, in comparison to 3D Simple Segmentation (Figure 5b) and 3D Spot Segmentation (Figure 5c), TOBLERONE ignores smaller objects arising from imaging artifacts. The algorithm automatically extracts a range of spatial statistics, allowing us to quantify the geometric properties of the cells. The available statistics include centroid coordinates, spread (standard deviation) in *x*-, *y*-, and *z-*directions, minimum, maximum, and mean intensity, and object volume (with the addition of object birth and death time for temporal). In this case, we have extracted the distribution of cell volumes (Figure 5e) for all 31 cells found and determined that the average volume of a Jurkat T-cell is 1961.6μm^3^, suggesting an average diameter of ~15.5 μm (assuming circularity). This is in accordance with existing literature^(^^49^ ^–^^51^ ^)^.

**Figure 6.**
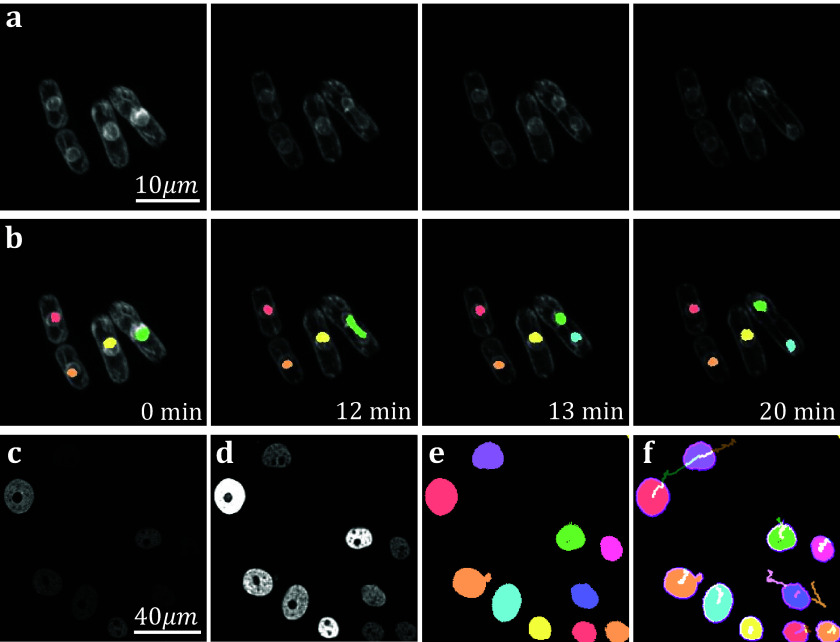
(a) Time series data of nuclear division in *S. pombe.* (b) Spatiotemporal segmentation of nuclei undergoing division. A nuclear division is recorded at 13 min. (c) Snapshot of GFP-GOWT1 mouse stem cell data. (d) Pre-processing improves background-foreground contrast. (e) Segmentation results from temporal TOBLERONE. (f) Track lines derived from applying Trackmate to segmentation.

The yeast *Schizosaccaramyces pombe*, expressing Bop1-mCherry, was cultivated and genetically modified using standard protocols, with the bop1 gene tagged with mCherry through homologous recombination^(^^34^ ^)^. Subsequently, images were captured using a Zeiss Axiovert 200 M microscope equipped with an UltraView RS-3 confocal system, with image stacks obtained at 1-minute intervals. The *S. pombe* cells were imaged in sealed growth chambers containing 2% agarose YES medium, and z-stack maximum projection images were processed using ImageJ. Images were first separated into distinct channels to distinguish nuclei from cell membranes. Segmentation was conducted on the underlying nuclear dye channel (taken at 600–710 nm wavelength light in accordance with mCherry emission spectrum) to discriminate nuclear envelopes from cell plasma membranes and promote a clearer segmentation result. Analysis on video data shows that temporal TOBLERONE can identify and track biological structures across multiple frames (Figure 6a–b). In addition, we can isolate instances in which connectivity was lost among biological structures, brought about by processes such as nuclear division (Figure 6b). Furthermore, we automatically extract statistics about the dynamic behaviors of the objects identified, such as the birth and death frame. Results suggest that an additional component was generated between the 12^th^ and 13^th^ minute of acquisition. From this, we can infer that the progression from telophase to interphase in the nuclear division of *S. pombe* can occur in under a minute, in line with existing reports^(^^52^^)^. Furthermore, we apply temporal TOBLERONE to GFP-GOWT1 mouse stem cell data (Figure 6c), acquired with a Leica TCS SP5 using a Plan-Apochromat 63x/1.4 (oil) objective lens, available from the Cell Tracking Challenge^(^^53^^,^^54^^)^. Following pre-processing (Figure 6d), results show good segmentation with clear background separation (Figure 6e). This output can be fed directly into track analysis software (Figure 6f), such as Trackmate, to highlight cell dynamics^(^^37^^,^^38^^)^. Ultimately, this suggests that topological video analysis may be a viable avenue for tracking cell movement and mitotic processes.

## 4. Discussion

Image segmentation is essential for automated and objective analysis of microscopy data. The advent of topological image analysis techniques allows for general image segmentation that is independent of the underlying geometry of the fluorescing objects. We have shown here that the principles of TDA can be extended to three dimensions both volumetrically and spatiotemporally. Applications to simulated data show that 3D TOBLERONE outperforms existing classical models and produces segmentations that are quantifiably more precise. Additionally, applications to experimental data reveal qualitative benefits of employing TDA over pre-existing geometric analysis, allowing for identification of more complex cell morphologies and more accurate segmentation. This allowed us to quantify the geometric properties of Jurkat T-cells, and in particular, that they express an average diameter of approximately ~15.5 μm.

Furthermore, we show that temporal TOBLERONE serves as a promising, novel technique for video decomposition in live-cell fluorescence microscopy. In the absence of alternative, classical video segmentation techniques, we demonstrate that temporal TOBLERONE alone provides a promising avenue for video decomposition and analysis in fluorescence microscopy. This allows for generic image segmentation and quantification of arbitrarily shaped biological structures, at high sensitivity and specificity, all while incorporating cell tracking across a range of frames. Moreover, we automatically extract statistics on the dynamic processes of the underlying objects, including the change in number of objects identified over time. In the context of fluorescence microscopy, this allows for automated extraction of temporally descriptive statistics such as the proliferation rate of cells or drift.

TOBLERONE presents a range of benefits over existing segmentation techniques. The runtimes of 3D and temporal TOBLERONE are on-par with existing 2D machine-learning methods for stacks with high signal-to-noise ratios. All simulated data sets, each comprised of 256 by 256 pixel images with up to 30 frames each, took no longer than ~2 minute to segment on a single processor^(^^12^ ^,^^13^ ^)^. In addition, both forms of TOBLERONE can be employed on any generic identifiable structures, not just cells. In the context of fluorescence microscopy, this could include any arbitrary biological structure that can be stained with fluorophores, such as organelles, vesicles, and the cytoskeleton^(^^55^ ^,^^56^ ^)^. For video segmentation, the roles of binarization, segmentation, and tracking will typically be delegated to distinct algorithms within a pipeline, which itself depends on user preferences and the extent of parameterization. However, TOBLERONE overcomes this limitation by encompassing all aspects of the pipeline in a single approach. As TDA techniques, 3DTOB and tempTOB allow for segmentation of arbitrarily shaped structures and are not impacted by geometric properties such as volume or morphology – as such, no geometric parameters need to be defined^(^^57^ ^)^. Additionally, cells that display a high degree of between-cell variation in morphologies can also be segmented. As such, TOBLERONE is highly applicable to data sets with isolated but highly non-convex biological structures. As a novel cell tracking technique, temporal TOBLERONE is capable of identifying when objects have split or merged and can therefore trace objects through connectivity rather than simply assigning each object per frame to the next closest object in subsequent frames.

To achieve a clean segmentation of an image, pre-processing techniques must usually be implemented to highlight the distinct topological structures, which can then be identified by TOBLERONE. Additionally, post-processing techniques may be necessary to filter out unwanted structures picked up by the algorithm, such as dead cells. However, these drawbacks are typical of most image segmentation algorithms. Each variant of TOBLERONE requires one parameter, which must sometimes be determined iteratively if image quality is poor. That said, topological features are inherently robust to variations in intensity in both 2D and 3D data, so a range of persistence thresholds will typically suffice^(^^58^ ^,^^59^ ^)^. It has been shown that topological segmentation is consistently accurate across varied intensity conditions provided there is sufficient contrast between the foreground and background^(^^28^ ^)^. As discussed, TOBLERONE is invariant of object morphology, which makes the algorithm extremely generalizable, but can lead to difficulties when separating densely-packed structures^(^^57^ ^,^^59^ ^)^. However, this typically imposes a consistent geometry upon cells, which can be exploited by subjecting each image to geometric post-processing through, for example, the Watershed algorithm, which specializes in isolating convex structures from binary images^(^^47^ ^)^. The tempTOB approach is not appropriate for data with low temporal resolution, as objects that are too far apart across frames may not be connected – in particular, if a cell drifts more than its own length across one frame. However, this is not common for conventional fluorescence microscopy.

The primary drawback of TOBLERONE is that the capacity to distinguish adjacent objects brings both additional computational complexity and greater data storage requirements. Since each object is considered separately, a distinct data structure must be created for every object found, regardless of the size of the object. During pre-processing, a contrast sweep discards background pixels by filtering out the lowest 5% of intensities. This greatly improves runtime in images with good contrast, but in cases where signal-to-noise ratio is high, individual background pixels may be segmented. In these images, there may be a significant increase in runtime and loss of segmentation quality. In addition, runtime increases quadratically with image dimensions due to the non-linear increase in pixel number. As such, we recommend sub-setting larger fields of view into smaller, more manageable ROIs (256 × 256 pixels or less where possible) and using appropriate pre-processing techniques such as smoothing and contrast enhancement.

In summary, we have shown here that 3D extensions of existing topological image analysis techniques can produce accurate and precise segmentations of images derived from fluorescence microscopy. As a subset of TDA, these algorithms can probe stacks of images containing arbitrarily shaped biological structures of any underlying topology. This allows for automated identification and segmentation of stainable cellular components which is fully reproducible. Furthermore, we can quantify spatial and temporal statistics allowing for automated tracking of volumetric and dynamic properties of biological structures.

## Data Availability

Simulated and experimental images are available at https://github.com/lucapanconi/toblerone. The data that support the findings of this study are available from the corresponding author upon reasonable request.
